# AcmB Is an S-Layer-Associated β-*N*-Acetylglucosaminidase and Functional Autolysin in Lactobacillus acidophilus NCFM

**DOI:** 10.1128/AEM.02025-16

**Published:** 2016-08-30

**Authors:** Brant R. Johnson, Todd R. Klaenhammer

**Affiliations:** Graduate Program in Microbiology, Department of Food, Bioprocessing and Nutrition Sciences, North Carolina State University, Raleigh, North Carolina, USA; Pennsylvania State University

## Abstract

Autolysins, also known as peptidoglycan hydrolases, are enzymes that hydrolyze specific bonds within bacterial cell wall peptidoglycan during cell division and daughter cell separation. Within the genome of Lactobacillus acidophilus NCFM, there are 11 genes encoding proteins with peptidoglycan hydrolase catalytic domains, 9 of which are predicted to be functional. Notably, 5 of the 9 putative autolysins in L. acidophilus NCFM are S-layer-associated proteins (SLAPs) noncovalently colocalized along with the surface (S)-layer at the cell surface. One of these SLAPs, AcmB, a β-*N*-acetylglucosaminidase encoded by the gene *lba0176* (*acmB*), was selected for functional analysis. *In silico* analysis revealed that *acmB* orthologs are found exclusively in S-layer- forming species of Lactobacillus. Chromosomal deletion of *acmB* resulted in aberrant cell division, autolysis, and autoaggregation. Complementation of *acmB* in the Δ*acmB* mutant restored the wild-type phenotype, confirming the role of this SLAP in cell division. The absence of AcmB within the exoproteome had a pleiotropic effect on the extracellular proteins covalently and noncovalently bound to the peptidoglycan, which likely led to the observed decrease in the binding capacity of the Δ*acmB* strain for mucin and extracellular matrices fibronectin, laminin, and collagen *in vitro*. These data suggest a functional association between the S-layer and the multiple autolysins noncovalently colocalized at the cell surface of L. acidophilus NCFM and other S-layer-producing Lactobacillus species.

**IMPORTANCE** Lactobacillus acidophilus is one of the most widely used probiotic microbes incorporated in many dairy foods and dietary supplements. This organism produces a surface (S)-layer, which is a self-assembling crystalline array found as the outermost layer of the cell wall. The S-layer, along with colocalized associated proteins, is an important mediator of probiotic activity through intestinal adhesion and modulation of the mucosal immune system. However, there is still a dearth of information regarding the basic cellular and evolutionary function of S-layers. Here, we demonstrate that multiple autolysins, responsible for breaking down the cell wall during cell division, are associated with the S-layer. Deletion of the gene encoding one of these S-layer-associated autolysins confirmed its autolytic role and resulted in reduced binding capacity to mucin and intestinal extracellular matrices. These data suggest a functional association between the S-layer and autolytic activity through the extracellular presentation of autolysins.

## INTRODUCTION

Beneficial microorganisms such as probiotics are defined by the FAO/WHO as “live microorganisms that, when administered in adequate amounts, confer a health benefit on the host” (1). Lactobacillus acidophilus NCFM is a generally recognized as safe, industrially significant lactic acid bacterium which has been sold commercially and consumed in various probiotic food formulations for more than 35 years (2). Predicated by the availability of a fully sequenced and annotated genome (3), L. acidophilus NCFM is one of the most studied and well-characterized probiotic bacteria (2, 4–8). Most notably, the probiotic activity of L. acidophilus is mediated by cell surface-associated components which interact with the host gastrointestinal mucosa and immune system (9–11).

As in other Gram-positive bacteria, the cell envelope of L. acidophilus is characterized by a lipid membrane surrounded by a thick peptidoglycan sacculus with a complex assemblage of macromolecules, including teichoic acids, polysaccharides, and proteins (12, 13). The peptidoglycan is composed of glycan chains consisting of alternating *N*-acetylglucosamine and *N*-acetylmuramic acid, linked via β-1,4 bonds and covalently cross-linked with peptide chains. Among the numerous functions of peptidoglycan are the maintenance of cell shape (14), integrity from osmotic pressure (15), and the presentation of proteins (12). Some of these proteins are covalently linked to the peptidoglycan via sortase and LPXTG motif recognition (16), while many others, including proteins which comprise the surface (S)-layer, are noncovalently attached through cell wall binding domains (CWBD) (17–19).

S-layers are semiporous, proteinaceous crystalline arrays consisting of self-assembling (glyco)protein subunits called S-layer proteins (SLPs). While S-layers can be found in all prokaryotes, including Gram-positive and Gram-negative organisms and many species of Archaea, S-layers are not ubiquitous to all microorganisms (17). In L. acidophilus, the S-layer monolayer is composed of a dominant protein constituent, SlpA (46 kDa), with minor constituents SlpB (47 kDa) and SlpX (51 kDa) (20). Because the S-layer is presented as the outermost layer of proteins on the cell wall, it has been the target for functional analysis of probiotic-host interactions. In fact, *in vitro* studies using intestinal epithelial cell lines suggest that the S-layer is a major factor in intestinal adhesion for L. acidophilus (21, 22). Furthermore, SlpA of L. acidophilus NCFM has been shown to bind dendritic cell C-type lectin receptors (23) and exert regulatory signals which mitigate inflammatory disease states and promote maintenance of healthy intestinal barrier function (24).

Despite the apparent importance of the S-layer for probiotic-host interactions, there is still a great deal that is not known about the composition and evolutionary function of S-layers. Complete functional analysis of the S-layer in L. acidophilus has been limited due to the apparent essentiality of the S-layer for cell survival and the ensuing difficulty of creating a stable deletion mutant of SlpA in L. acidophilus (Y. J. Goh and T. R. Klaenhammer, unpublished data). Exoproteomic analysis of L. acidophilus NCFM and other S-layer-forming lactobacilli has revealed the presence of numerous S-layer-associated proteins (SLAPs), which are colocalized with the S-layer through noncovalent association with the cell wall peptidoglycan (10, 25). In addition to uncharacterized proteins with putative probiotic activity, numerous autolysins were found in these SLAP fractions (10, 25).

Autolysins, also known as peptidoglycan hydrolases (PGH), are a class of enzymes responsible for peptidoglycan turnover during cell division and daughter cell separation (13, 26, 27). PGH have numerous catalytic domains and are normally bound to the cell wall through LysM- or SH3-anchoring domains (28, 29). Notably, the PGH identified in the SLAP fractions of L. acidophilus NCFM and other S-layer-forming Lactobacillus species are anchored to the cell wall with noncovalent attachment domains (NCAD) (pfam03217), the same domains found in SLPs (10, 25, 30). In this study, an S-layer-associated β-*N*-acetylglucosaminidase autolysin, designated AcmB, was selected for functional analysis in L. acidophilus NCFM. Chromosomal deletion of *acmB* resulted in aberrant cell division, autolysis, and autoaggregation, confirming the role of this SLAP in PGH activity. Further, the absence of AcmB within the exoproteome had a pleiotropic effect on cell surface proteins associated with the peptidoglycan, as measured through the reduced ability of the Δ*acmB* strain to bind to mucin and extracellular matrices *in vitro*. Here we present the S-layer as a scaffold for numerous proteins, including autolysins. Analysis of these S-layer-associated autolysins will undoubtedly lead to a more comprehensive understanding of the evolutionary function of the S-layer in S-layer-forming species of Lactobacillus.

## MATERIALS AND METHODS

### Bacterial strains and growth conditions.

The bacterial strains, plasmids, and primers used in this study are listed in Table 1. L. acidophilus strains were propagated in de Man Rogosa Sharpe (MRS) broth (Difco) under aerobic conditions, statically or on MRS solid medium containing 1.5% (wt/vol) agar (Difco) under anaerobic conditions at 37°C and at 42°C where indicated. Recombinant strains were selected in the presence of 2 μg/ml erythromycin (Sigma-Aldrich, St. Louis, MO) and/or 2 to 5 μg/ml chloramphenicol (Sigma). Escherichia coli strains were grown in brain heart infusion (Difco) medium at 37°C with aeration. E. coli EC101 was grown in the presence of 40 μg/ml kanamycin (Sigma-Aldrich), while NCK1911 and transformants were grown with 40 mg kanamycin and 150 μg/ml erythromycin. Counterselection of L. acidophilus plasmid-free excision recombinants was performed using 5-fluorouracil-supplemented glucose semidefined medium, as previously described (20).

**TABLE 1:** Strains, plasmids, and primers used in this study

Strain, plasmid, or primer	Genotype or characteristic(s)^*a*^	Reference or source
L. acidophilus strains		
NCFM	Human intestinal isolate	2
NCK1909	NCFM carrying a 315-bp deletion within the *upp* gene	20
NCK1910	NCK1909 harboring pTRK669; host for pORI-based counterselective integration vector	20
NCK2395	NCK1909 carrying a 1,103-bp deletion within the *lba0176* gene	This study
NCK2397	NCK2395 harboring pTRK1098 for complementation of *lba0176*	This study
E. coli strains		
NCK1831	EC101 host for pORI-based plasmids	58
NCK1911	Host harboring pTRK935, Kn^r^, Em^r^	20
NCK2394	Host harboring pTRK1097, Kn^r^, Em^r^	This study
NCK2396	Host harboring pTRK1098, Em^r^	This study
Plasmids		
pTRK669	Ori (pWV01), Cm^r^, RepA^+^ thermosensitive	59
pTRK935	pORI *upp*-based counterselective integration vector	20
pTRK882	Δ*cat* derivative of pGK12 with MCS from pORI28 and cloned P*_pgm_*, Em^r^	40
pTRK1097	pTRK935 with flanking regions of *lba0176* cloned into BamHI/SacI site	This study
pTRK1098	pTRK882 with *lba0176* cloned into EcoRI/BamHI site	This study
Primers^*b*^		
Construction of Δ*acmB* mutant		
0176BamHIF	GTAATAGGATCCATCTGAGTTGTTTGGTAATG	This study
0176R	CATTATTCACTACTGGGGTA	This study
0176Soe	TACCCCAGTAGTGAATAATGTATTACAGAATCGGTATTCG	This study
0176SacIR	TAAAGTAGAGCTCGCATCATTGTTAATTGATTT	This study
Screening of Δ*acmB* locus		
0176up	AACCAAAGTTAAATGAAACA	This study
0176dw	CTTAGCTTGCAAATCATAGT	This study
Complementation of *acmB*		
C0176EcoRIF	GATCGAATTCAA*GGAGA*ACGTAT**ATG**AAGAAGAGACTTTTGACCCAGC	This study
C0176BamHIR	GATCGGATCCTACATGAAGTTAGCTTTTTTAATG	This study

a Km, kanamycin; Em, erythromycin; Cm, chloramphenicol.

b Restriction sites are underlined. Ribosome binding sites are in italics. Start codons are in bold.

### DNA manipulation and transformation.

Genomic DNA from L. acidophilus strains was isolated using a Fungal/Bacterial DNA MiniPrep kit (Zymo Research). Plasmid DNA from E. coli was isolated using a QIAprep Spin Miniprep kit (Qiagen). Restriction enzyme digestions and ligations were performed using Roche restriction enzymes (Roche Diagnostics) and T4 DNA ligase (New England BioLabs), respectively. PCR primers were designed based on the genomic sequence data and synthesized by Integrated DNA Technologies. PCRs were carried out in Bio-Rad MyCycler thermocyclers (Bio-Rad Laboratories) using Choice-*Taq* Blue DNA polymerase (Denville Scientific) for screening of recombinants and *PfuUltra* II fusion HS DNA polymerase (Agilent Technologies) for cloning purposes. PCR amplicons were analyzed on 0.8% agarose gels and purified using QIAquick gel extraction kits (Qiagen).

E. coli EC101 cells were made competent using a rubidium chloride competent cell protocol (31). L. acidophilus cells were prepared for electrotransformation using a modified penicillin treatment protocol (20, 32, 33).

### *In silico* and RNA sequencing analyses.

Predicted peptidoglycan hydrolases were identified from the L. acidophilus NCFM genome (3) (NCBI accession number NC_006814). Homologous sequences were identified and compared using the BLASTn and BLASTp features of NCBI, as well as SANSparallel (34). Signal peptidase cleavage sites for protein sequences were predicted using SignalP 4.1 (35). Protein domains were identified using UniProt and the Pfam protein family database (36, 37). RNA sequencing analysis from a previous study (10) was utilized to examine mRNA expression of the predicted peptidoglycan hydrolases. Gene expression was measured by the normalized transcripts per million (TPM) calculator within Geneious 8.0.5 (38).

### Deletion of *acmB* in L. acidophilus NCFM.

The *upp*-based counterselection gene replacement method (20) was used to create an internal deletion of 1,103 bp in *acmB* (*lba0176*) of NCK1909, a *upp*-deficient background strain of L. acidophilus NCFM. With splicing by overlap extension PCR (39), the 1-kb regions flanking the deletion target were spliced with a BamHI restricted site added at the upstream end and SacI at the downstream end. This construct was digested with BamHI and SacI and then ligated into the polylinker of the similarly digested integration plasmid pTRK935 and transformed into competent E. coli EC101. The resulting recombinant plasmid, pTRK1097, was transformed into L. acidophilus NCK1909 harboring the helper plasmid pTRK669 (NCK1910). Single crossover integrants were screened as described previously (20). Colonies with the Δ*acmB* genotype were screened by PCR among the double recombinants recovered on glucose semidefined medium agar plates containing 5-fluorouracil. Deletion of *acmB* was confirmed by PCR and sequencing, and the resulting Δ*acmB* mutant was designated NCK2395.

### Complementation of *acmB* in Δ*acmB* strain of L. acidophilus NCFM.

The Δ*acmB* strain of L. acidophilus NCFM was complemented using pTRK882, an expression plasmid with the promoter for *pgm* (*lba0185*), encoding a phosphoglyceromutase (40). The *acmB* gene, along with its native ribosome binding site, was amplified with EcoRI and BamHI restriction sites added to the 5′ and 3′ ends of the amplicon and subsequently cloned into the polylinker of pTRK882. The integrity of the insert was confirmed by DNA sequencing. The resulting recombinant plasmid, pTRK1098, was electroporated into the Δ*acmB* mutant of L. acidophilus NCFM. Transformants were selected by erythromycin resistance, generating NCK2397 for phenotypic comparison.

### LiCl extraction of SLAPs.

Noncovalently bound cell surface proteins, including S-layer proteins and S-layer-associated proteins (SLAPs), were extracted from NCK1909 and Δ*acmB L. acidophilus* NCFM strains using LiCl denaturing salt, as described previously (25). Proteins were quantified via a bicinchoninic acid assay kit (Thermo Scientific) and visualized via SDS-PAGE using precast 4 to 20% Precise Tris-HEPES protein gels (Thermo Scientific). The gels were stained using AcquaStain (Bulldog Bio) according to the instructions of the manufacturer.

### Microscopic and morphological assessments.

Morphological assessment of L. acidophilus NCFM strain was performed using a phase-contrast light microscope at ×40 magnification (Nikon Eclipse E600). Cells were observed over a growth period of 24 h in MRS broth at 37°C or MRS broth with 5 μg/ml erythromycin for the complemented strain NCK2397. Pictures were taken using a QImaging MicroPublisher 5.0 RTV camera attachment at 1-, 4-, 7-, 14-, and 24-h time points. Cell chain length was measured using Image-Pro Insight software (Media Cybernetics).

### Autoaggregation and autolysis assays.

Autoaggregation assays were performed as described previously (41). Bacteria were grown in MRS broth with 5 μg/ml erythromycin where necessary for 16 h, harvested by centrifugation at 1,771 × *g* for 10 min, and washed twice with phosphate-buffered saline (PBS) (pH 7.4). Washed cells were resuspended in PBS to an adjusted optical density at 600 nm (OD_600_) of 1. Cell suspensions were mixed by vortexing for 10 s, and autoaggregation was determined over 5 h at room temperature. Every hour, 100 μl of the upper suspension was transferred to a cuvette with 900 μl PBS, and the absorbance (OD_600_) was measured. Autoaggregation percentages were calculated as follows: 1 − (*A_t_*/*A*_0_) × 100, where *A_t_* is the OD_600_ at time (*t*) = 1, 2, 3, 4, and 5 h and *A*_0_ is the OD_600_ at time zero.

Autolysis was performed as described previously (29) with some modifications. L. acidophilus NCFM strains were grown to late-exponential phase (OD_600_ of ∼1.0) and were harvested via centrifugation at 1,771 × *g* for 10 min. Cells were washed once with PBS (pH 7.4) and resuspended in PBS (pH 7.4) supplemented with 0.05% Triton X-100. Suspensions were transferred to sterile 96-well plates with transparent bottoms. OD_600_ values was assessed every 20 min for 24 h using a FLUOStar Optima microtiter plate reader (BMG Technologies) at 37°C. Autolysis was calculated as the decrease in OD_600_ relative to the initial OD_600_ at time zero.

### Mucin and ECM adherence assays.

Extracellular matrix (ECM) binding assays were performed as described previously (42). Mucin (type III from porcine stomach; Sigma) was dissolved in PBS to a final concentration of 10 mg/ml. Fibronectin (from human plasma; Sigma), collagen (type IV from human cell culture; Sigma), and laminin (from Engelbreth-Holm-Swarm murine sarcoma/basement membrane; Sigma) were diluted in 50 mM carbonate-bicarbonate buffer (pH 9.6) (Sigma) to a final concentration of 10 μg/ml. For each assay, a Nunc MaxiSorp 96-well microplate (Sigma) was coated with 100 μl/well substrate and incubated at 4°C overnight. The wells were washed twice with PBS to remove excess substrate before blocking with 150 μl/well 2% bovine serum albumin (BSA) solution (Sigma) for 2 h at 37°C. Excess BSA was removed by two washes with PBS (pH 7.4).

Bacterial cells were grown in MRS broth to stationary phase (16 h) in preparation for the assay. Cultures were centrifuged (1,771 × *g*, 15 min, room temperature), washed once, and resuspended in PBS (pH 4.75). Cell density was adjusted to ∼1 × 10^8^ CFU/ml based on previously calculated OD_600_/CFU ratios. Cell suspensions (100 μl) were added to each mucin or ECM-coated well. Initial cell counts were enumerated on MRS agar plates. After incubation for 1 h at 37°C, the wells were gently washed five times with 200 μl/well PBS. Adhered cells were recovered by adding 100 μl of 0.05% Triton X-100 solution (FisherBiotech) (prepared in PBS) to each well and agitating on an orbital shaker (200 rpm) for 15 min. Cell suspensions were transferred into 900 μl of 0.1× MRS before being further diluted and plated in duplicate on MRS plates. Colonies were enumerated and expressed as a percentage of relative adherence (mutant CFU/parent CFU), where parent (NCK1909) CFU were defined as 100%.

## RESULTS

### *In silico* analysis of autolysins within L. acidophilus NCFM.

**FIG 1:**
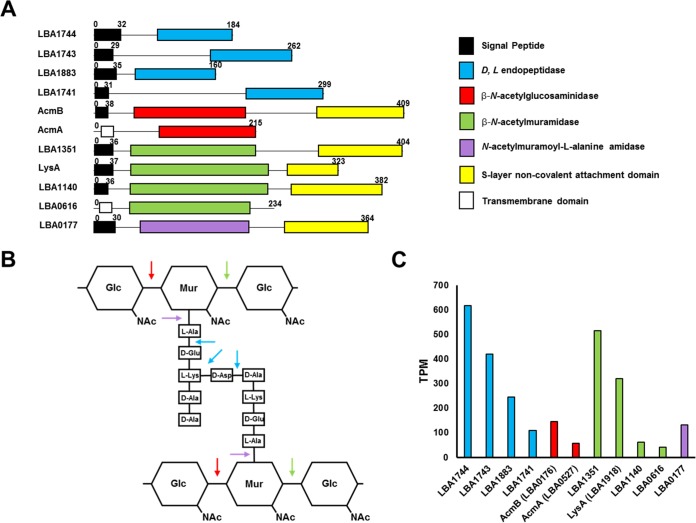
(A) The modular domain organization of the 11 predicted autolysins of L. acidophilus NCFM. The numbers indicate the corresponding amino acid length. (B) The corresponding specific activity of each catalytic domain on the peptidoglycan structure. (C) RNA-seq transcriptional analysis of the gene encoding each autolysin, with colors corresponding to mRNA transcripts from genes with the following catalytic activities: blue, endopeptidase; red, β-*N*-acetylglucosaminidase; green, β-*N*-acetylmuramidase; and purple, *N*-acetylmuramoyl-l-alanine amidase.

Within the genome of L. acidophilus NCFM, there are 11 genes which encode putative autolysins with predicted PGH activity (Table 2; Fig. 1). These PGH activities can be subdivided into four classes: d- and l-endopeptidases (pfam00877) (Fig. 1A, blue), β-*N*-acetylglucosaminidases (pfam01832) (Fig. 1A, red), β-*N*-acetylmuramidases (pfam01183) (Fig. 1A, green), and *N*-acetylmuramoyl-l-alanine amidases (pfam01510) (Fig. 1A, purple). The four classes of autolysins predicted in L. acidophilus have the required specificities to hydrolyze all components of the peptidoglycan (Fig. 1B). Further, there is apparent redundancy in three of the PGH classes, with four endopeptidases, two β-*N*-acetylglucosaminidases, and four β-*N*-acetylmuramidases encoded within the genome (Fig. 1A). With transcriptome sequencing (RNA-seq) data from a previous study (10), mid-logarithmic phase transcriptional profiles of the predicted autolysins were compared to analyze which may be the primary autolysin of each class (Fig. 1C). Based on these data, LBA1744, AcmB, and LBA1351 were found to be the most highly expressed endopeptidase, β-*N*-acetylglucosaminidase, and β-*N*-acetylmuramidase, respectively (Fig. 1C). LBA0177 appears to be the sole *N*-acetylmuramoyl-l-alanine amidase of L. acidophilus NCFM. Two genes with very low expression, AcmA and LBA0616, encoding a β-*N*-acetylglucosaminidase and a β-*N*-acetylmuramidase, do not have predicted signal peptide sequences and thus are likely not functional autolysins (Fig. 1A and C).

**TABLE 2:** Autolysins of L. acidophilus NCFM

Open reading frame	Identification	UniProt code	PGH domain^*a*^	Amino acid length	SignalP^*b*^	SLAP^*c*^
Endopeptidase						
*lba1744*	Putative glycosidase	Q5FIB9	pfam00877	184	+	−
*lba1741*	Cell wall-associated hydrolase	Q5FIC1	pfam00877	299	+	−
*lba1743*	Cell wall-associated hydrolase	Q5FIC0	pfam00877	262	+	−
*lba1883*	NLP-P60 secreted protein	Q5FHZ1	pfam00877	160	+	−
β-*N*-Acetylglucosaminidase						
*acmA* (*lba0527*)	*N*-Acetylglucosaminidase	Q5FLL6	pfam01832	215	−	−
*acmB* (*lba0176*)	*N*-Acetylglucosaminidase	Q5FMJ9	pfam01832	409	+	+
β-*N*-Acetylmuramidase						
*lba0616*	Putative uncharacterized protein	Q5FLC9	pfam01183	234	−	−
*lysA* (*lba1918*)	Lysin	Q5FHV9	pfam01183	323	+	+
*lba1140*	Lysin	Q5FJZ4	pfam01183	382	+	+
*lba1351*	Lysin	Q5FJE6	pfam01183	404	+	+
*N*-Acetylmuramoyl-l-alanine amidase						
*lba0177*	Autolysin/amidase	Q5FMJ8	pfam01510	364	+	+

a Catalytic domain determined by pfam (37).

b SignalP, signal peptidase cleavage site (35).

c Previously identified as an S-layer-associated protein (25).

Notably, five of the nine functional autolysins have been identified as SLAP constituents (Table 2) of the noncovalently bound exoproteome in L. acidophilus NCFM and other S-layer-forming species of Lactobacillus acidophilus homology group (10, 25). In fact, these proteins have the noncovalent attachment domain (NCAD) (pfam03217) found in other SLP, suggesting their colocalization at the cell surface along with the S-layer (Fig. 1A, yellow). These SLAPs encompass all of the predicted functional β-*N*-acetylmuramidases, as well as the sole *N*-acetylmuramoyl-l-alanine amidase, LBA0177, and the β-*N*-acetylglucosaminidase, AcmB. The four endopeptidases were not found in the SLAP fractions of the previous studies and do not have the NCAD domains (Fig. 1A). AcmB was selected for functional analysis based on its prevalence in the noncovalently bound exoproteome and conservation as a SLAP in L. acidophilus NCFM and other S-layer-forming species of the homology group (see below).

### *In silico* genomic analysis of AcmB.

Although the gene encoding AcmB (*lba0176*) was previously annotated as a β-*N*-acetylmuramidase (3), the protein has a mannosyl-glycoprotein endo-β-*N*-acetylglucosaminidase catalytic domain (pfam01832), suggesting that AcmB is a β-*N*-acetylglucosaminidase. Within the chromosome of L. acidophilus NCFM, *acmA* (*lba0527*) is the only other gene which also contains the β-*N*-acetylglucosaminidase domain (Fig. 1A). However, *acmA* may be truncated, does not have a signal peptide sequence (Fig. 1A), and demonstrates reduced transcriptional expression compared to that of *acmB*, which does contain a signal peptide sequence (Fig. 1C). For these reasons, AcmB appears to be the primary β-*N*-acetylglucosaminidase for L. acidophilus NCFM.

**FIG 2:**
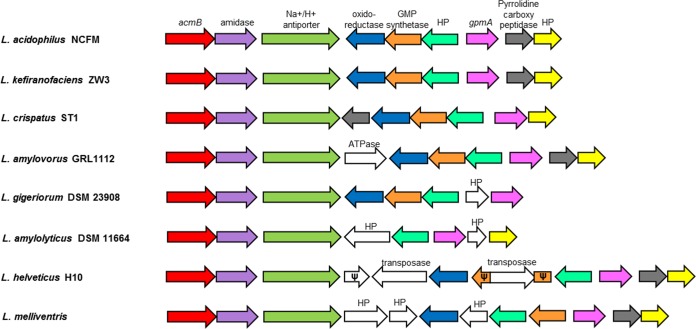
*acmB* orthologs were found in various S-layer-forming strains of Lactobacillus, including *L. amylovorus* GRL1112, L. helveticus H10, *L. kefiranofaciens* ZW3, L. crispatus ST1, *L. melliventris*, *L. amylolyticus* DSM 11664, and *L. gigeriorum* DSM 23908. The genetic region surrounding *acmB* was highly syntenic in all species examined. Arrows represent genes, while the colors represent specific genes, as follows: red, *acmB*; purple, amidase; green, Na^+^/H^+^ ion transporter; blue, oxidoreductase; orange, GMP synthetase; teal, conserved hypothetical protein (HP); pink, *gpmA*; dark gray, pyrrolidine carboxypeptidase; and yellow, conserved HP. Genes in white are divergent genes unique to each species where indicated. Ψ indicates a truncated pseudogene.

AcmB orthologs are found in numerous Lactobacillus species, including *L. amylovorus* GRL1112 (81.7% amino acid identity), L. helveticus H10 (78.5%), *L. kefiranofaciens* ZW3 (75.8%), L. crispatus ST1 (73.3%), *L. melliventris* (61%), *L. amylolyticus* DSM 11664 (61.3%), and *L. gigeriorum* DSM 23908 (59.3%). Most notably, the AcmB orthologs are found exclusively in S-layer-forming species of Lactobacillus, providing further evidence that AcmB is an S-layer-associated protein. There are additional orthologs found in the genomic region surrounding AcmB in these S-layer-forming species, compared to those in L. acidophilus NCFM (Fig. 2). In fact, the genomic region directly downstream of *acmB* appears to be syntenic and conserved among the seven S-layer-forming species listed above (Fig. 2). Among the orthologs with synteny in this region are genes encoding an S-layer-associated *N*-acetylmuramoyl-l-alanine amidase, an uncharacterized Na^+^/H^+^ ion transporter, an oxidoreductase, a GMP synthetase, a phosphoglyceromutase, a pyrrolidine carboxypeptidase, and two hypothetical proteins (Fig. 2). These genes were all found to be in the genomic region surrounding AcmB in the S-layer-forming strains, with the exception of the pyrrolidine carboxypeptidase, which is absent in *L. amylolyticus* DSM 11664 and *L. gigeriorum* DSM 23908 (Fig. 2). In *L. kefiranofaciens* ZW3, the position of the genes within the region is identical to that in L. acidophilus NCFM and is likewise conserved in L. crispatus ST1, with the exception of the translocation of a pyrrolidine carboxypeptidase gene from the positive strand to the negative strand of the genome (Fig. 2).

### Deletion of *acmB* from the chromosome of L. acidophilus NCFM and corresponding complementation of *acmB* in the Δ*acmB* mutant.

**FIG 3:**
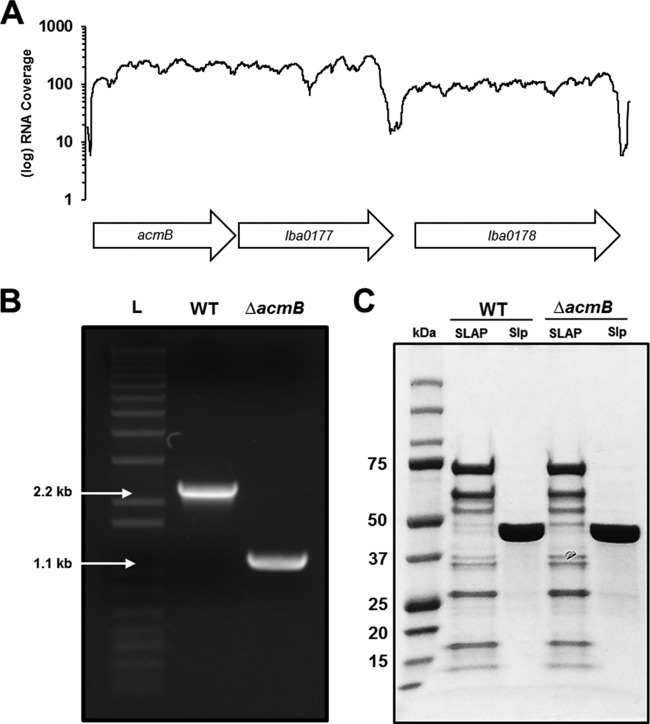
The gene encoding AcmB was deleted from the chromosome of L. acidophilus NCFM. (A) RNA-seq analysis demonstrates that *acmB* is polycistronically expressed with *lba0177*, which encodes an S-layer-associated *N*-acetylmuramoyl-l-alanine amidase. (B) Gel electrophoresis of PCR products using the primers indicated in panel A for the parent strain (WT) compared to the Δ*acmB* mutant. The deletion was confirmed by sequencing. L, DNA ladder. (C) SDS-PAGE of the noncovalently bound extracellular S-layer proteins (SLP) and S-layer-associated proteins (SLAPs) isolated from both the WT and Δ*acmB* strains.

With a *upp*-based counterselective gene replacement method (20), a markerless chromosomal deletion of *acmB* was made in a *upp*-deficient background host of L. acidophilus NCFM. The Δ*acmB* genotype was confirmed using PCR and DNA sequencing with primers flanking the 1,227-bp *acmB* gene (Fig. 3A and B). The absence of AcmB from the SLAP fraction of the Δ*acmB* strain was not observed visually by SDS-PAGE, due to multiple 45-kDa proteins in the fraction (Fig. 3C). Furthermore, the Δ*acmB* mutant did not appear to have an altered SLAP profile compared to that of the wild type (WT) (Fig. 3C). For phenotypic analysis, *acmB* was also complemented in the Δ*acmB* strain using pTRK1098, an expression plasmid with the constitutive *pgm* promoter.

### Phenotypic analyses of the Δ*acmB* mutant.

Following deletion confirmation, the Δ*acmB* strain was phenotypically assessed for (i) cell morphology, (ii) autoaggregation and autolytic capacity, and (iii) the ability to bind to mucin and extracellular matrices (ECM) collagen, fibronectin, and laminin.

### Cellular morphology of the Δ*acmB* mutant.

**FIG 4:**
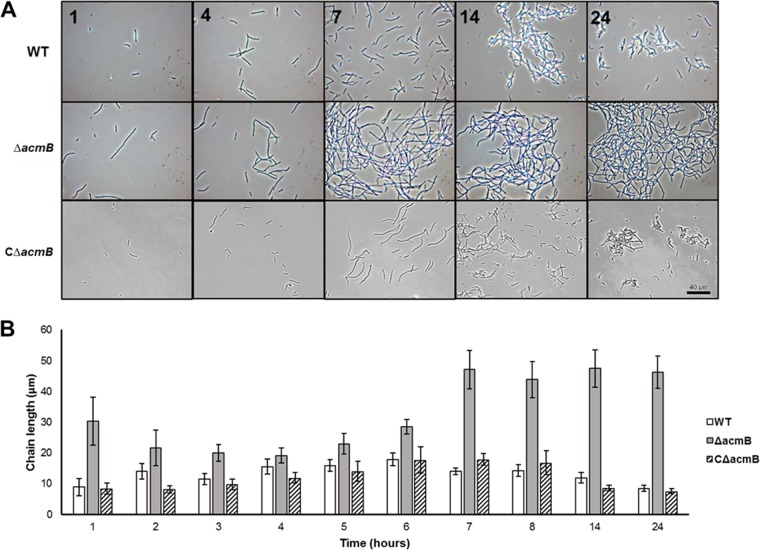
(A) The cellular morphologies of the wild-type (WT), mutant (Δ*acmB*), and *acmB* complemented (CΔ*acmB*) strains were assessed using phase-contrast light microscopy over a 24-h growth period. (B) Chain length measurements were taken for the WT, Δ*acmB*, and CΔ*acmB* strains. The chain length for the Δ*acmB* mutant (*n* = 611 cells) was significantly higher than those for the WT (*n* = 661 cells) and CΔ*acmB* (*n* = 316 cells) strains across all time points measured (*P* < 0.001). Error bars represent confidence intervals.

Cellular morphology of the Δ*acmB* strain was assessed by light microscopy over a 24-h growth time course in MRS culture (Fig. 4). Compared to the WT strain, the Δ*acmB* mutant had a distinctive morphological phenotype consisting of increased chain lengths and autoaggregation (Fig. 4A). Complementation of *acmB* in the Δ*acmB* strain (CΔ*acmB*) resulted in a return to WT morphology (Fig. 4A). This cell division-related morphology was quantified by measuring the chain lengths of dividing cells throughout the 24-h time course (Fig. 4B). Both the WT and CΔ*acmB* strains demonstrated prototypical chain lengths which followed a standard bacterial growth curve (Fig. 4B, white and striped bars). Specifically, chain lengths increased from lag phase to logarithmic phase (hours 1 to 6) and decreased as a result of dechaining during the transition to stationary phase (hours 7 to 24) (Fig. 4B). On the contrary, the Δ*acmB* mutant had a statistically significant increase in chain length across all measurements (*P* < 0.001) (Fig. 4B, gray bars). At the start of lag phase (hour 1), the Δ*acmB* strain had a pronounced increase in chain length (mean [M], 30.32 μm; confidence interval [CI] = 7.78) compared to those for the WT (M, 8.83 μm; CI, 2.77) and CΔ*acmB* (M, 8.34 μm; CI, 1.79) strains, likely due to residual cells from inoculation transfer of the previous stationary-phase culture (Fig. 4B). By early-logarithmic phase, the differences in chain lengths were less evident between the Δ*acmB* mutant and the WT and CΔ*acmB* strains. However, by mid-logarithmic phase (hour 7) the chain lengths in the Δ*acmB* culture were considerably longer (M, 47.0423 μm; CI, 6.17) than those for the WT (M, 13.94 μm; CI, 0.58) and CΔ*acmB* (M, 17.85 μm; CI, 1.91) strains (Fig. 4B), suggesting aberrant dechaining and daughter cell separation. For the remainder of the time course, the Δ*acmB* strain maintained increased chain lengths while the CΔ*acmB* and WT strains underwent normal cell division, as indicated by a concomitant decrease in cell chain lengths (Fig. 4B).

### Autoaggregation and autolysis of the Δ*acmB* mutant.

**FIG 5:**
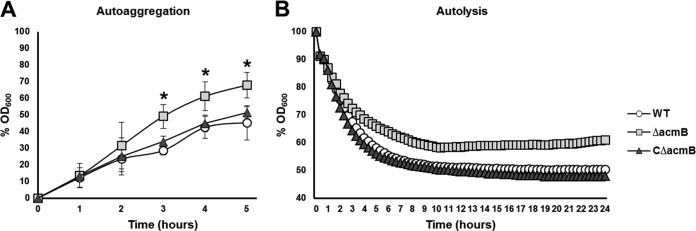
(A) Autoaggregation of WT, mutant (Δ*acmB*), and complemented (CΔ*acmB*) cells. Asterisks indicate statistical significance (*P* < 0.001). (B) Triton X-100-induced autolysis assays for WT, Δ*acmB*, and complemented strains. The differences between the Δ*acmB* mutant and the WT and complemented strains are statistically significant (*P* < 0.001). Each assay was performed in triplicate; all error bars represent confidence intervals.

Based on the abnormal cellular morphology and aberrant dechaining phenotype of the Δ*acmB* mutant, the autoaggregation and autolytic capacity of this strain were evaluated (Fig. 5). The sedimentation rates of the Δ*acmB*, WT, and complemented CΔ*acmB* strains were measured over 5 h in PBS (Fig. 5A). For the first 2 h, the three strains had comparable autoaggregation rates. By 3 h, the autoaggregation of the Δ*acmB* mutant (M, 49.24%; CI, 7.16%) was significantly higher (*P* < 0.001) than those of the WT (M, 28.69%; CI, 2.68%) and CΔ*acmB* (M, 33.94%; CI, 3.19%) strains (Fig. 5A). The differences were most pronounced at 5 h, at which the Δ*acmB* mutant had an autoaggregation percentage of 68.05% (CI, 7.73%), compared to 45.42% (CI, 10.35%) and 51.61% (CI, 3.31%) for the WT and CΔ*acmB* strains, respectively (*P* < 0.01) (Fig. 5A). To assess the autolytic behavior of the Δ*acmB*, WT, and CΔ*acmB* strains, Triton X-100-induced autolysis assays were performed (Fig. 5B). The rate of autolysis in the Δ*acmB* mutant was significantly lower than in the WT and complemented strains (*P* < 0.05). Specifically, Triton X-100-induced cells resulted in 40% autolysis of the Δ*acmB* population, compared to 50% in the WT and 52% in the CΔ*acmB* strain (Fig. 5B). These data demonstrate that the absence of AcmB in the Δ*acmB* strain causes an increase in autoaggregation, along with a decrease in stress-induced autolysis.

### Adherence capacity of the Δ*acmB* mutant.

**FIG 6:**
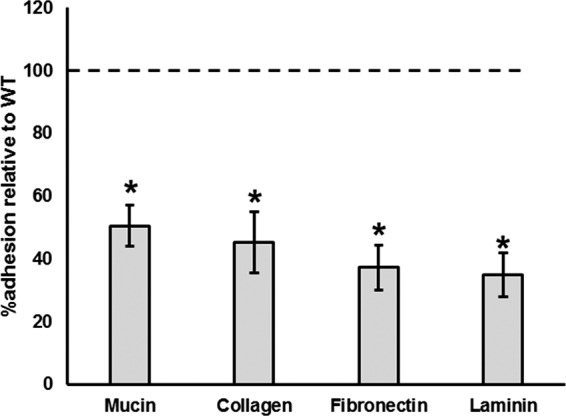
The ability of the Δ*acmB* mutant to bind to mucin and extracellular matrices (ECM) was assessed. Compared the wild-type (WT) reference (dotted line), the Δ*acmB* strain showed a significant reduction in binding to mucin, collagen, fibronectin, and laminin. Asterisks indicate statistical significance (*P* < 0.001). Adherence assays were performed in triplicate; all error bars represent confidence intervals.

Extracellular proteins localized to the cell surface are important mediators of probiotic activity, including adhesion to host intestinal epithelial mucus layer and ECM. Because of the irregular morphology of the Δ*acmB* strain, the ability of this mutant to bind mucin and ECM, including collagen, fibronectin, and laminin, was examined. The binding capacity of the Δ*acmB* mutant was significantly reduced relative to that of the WT for mucin and all ECM tested (Fig. 6). Specifically, there was a 50% reduction of cells bound to type III porcine mucin (*P* < 0.002), a 55% reduction of cells bound to type IV human collagen (*P* < 0.001), a 63% reduction of cells bound to human plasma fibronectin (*P* < 0.001), and a 65% reduction in adherence to murine laminin (*P* < 0.001), relative to those of the WT. These data suggest that the absence of AcmB has a pleiotropic effect on the cell surface, which results in decreased binding to various ECM.

## DISCUSSION

The Gram-positive cell wall is composed of a thick peptidoglycan sacculus responsible for sustaining cell shape, resistance against environmental and osmotic stresses, and the covalent and noncovalent presentation of proteins (26). Extracellular proteins responsible for the turnover of peptidoglycan during cell division and daughter cell separation are known as peptidoglycan hydrolases (PGH), or autolysins (27). These PGH are divided into four classes: (i) β-*N*-acetylmuramidases, (ii) β-*N*-acetylglucosamidases, (iii) *N*-acetylmuramoyl-l-amidases, and (iv) peptidases (28).

In this study, the PGH complement of L. acidophilus NCFM was identified and AcmB, an S-layer-associated β-*N*-acetylglucosaminidase, was functionally characterized. Within the genome of L. acidophilus NCFM, 11 genes encoding putative PGH were identified, including four β-*N*-acetylmuramidases, two β-*N*-acetylglucosaminidases, one *N*-acetylmuramoyl-l-amidase, and four peptidases. Nine of these PGH are predicted to be functional based on the presence of signal peptide sequences and RNA transcription analyses. The redundancy of encoded autolysins is consistent with the findings of previous studies identifying the PGH complement in other lactobacilli. L. casei BL23 encodes 13 PGH (43), while L. plantarum WCFS1 encodes 12 (29) and silage-fermenting *L. buchneri* CD034 encodes 24 (44). Notably, the closely related and S-layer-forming cheese-ripening bacterium L. helveticus DPC 4571 encodes 9 autolysins, including 5 orthologs from the PGH identified in this study (45). Redundancy of PGH within bacterial genomes is widespread (27) due to the essentiality of autolysin activity for cell survival (46). Further, this redundancy may be due to the fact that many of these hydrolases have more than one function. PGH of Bacillus subtilis have numerous described functions beyond autolytic hydrolase activity, including roles in protein turnover and secretion, motility, and competence (47). However, the characterization of autolysin activity in Lactobacillus species, to date, has been primarily focused on the hydrolysis of peptidoglycan sacculi (13).

Autolysins are associated with the cell wall via numerous cell wall binding domains (CWBD) including CHAP domains, GW domains, SH3 domains, and LysM domains (13, 27). Notably, in L. acidophilus, the primary CWBD is the S-layer noncovalent attachment domain (NCAD) (pfam03217), which is responsible for the noncovalent attachment of the S-layer and S-layer-associated proteins in Lactobacillus (10, 30). Five of the nine functional autolysins in L. acidophilus NCFM have the S-layer NCAD domain, suggesting extracellular colocalization with the S-layer. In fact, these five proteins were previously identified in the LiCl-purified SLAP fraction of the L. acidophilus NCFM noncovalently bound exoproteome (25). Based on the *in silico* prediction of the PGH complement identified in this study and the previously identified SLAPs (25), all encoded β-*N*-acetylmuramidases, β-*N*-acetylglucosaminidases, and *N*-acetylmuramoyl-l-alanine amidases with a signal peptide sequence in *L*. acidophilus NCFM appear to be SLAPs. These findings are supported by previously published studies on autolysin activity in the S-layer-forming strains L. helveticus ATCC 12046 and L. helveticus ISLC5, in which two autolysins were copurified with the S-layer using LiCl (48, 49). It is also notable that there are two autolysins described in the PGH complement of L. helveticus DPC 4571 which contain the S-layer NCAD domain (45).

One of the five S-layer-associated autolysins is AcmB, a predicted 45-kDa protein with a β-*N*-acetylglucosaminidase catalytic domain. Further evidence of the association between this autolysin and the S-layer can be seen through examination of the AcmB orthologs in Lactobacillus species. All known AcmB orthologs are found exclusively in S-layer-forming species of the L. acidophilus homology group. Notably, AcmB orthologs are not found in the closely related, but non-S-layer-forming members of the homology group, including *Lactobacillus gasseri*, *Lactobacillus johnsonii*, and the progenitor, Lactobacillus delbrueckii subsp. *bulgaricus*. These data are supported by our recent exoproteomic survey of S-layer- and non-S-layer-forming species of the L. acidophilus homology group (10). Autolysins, including AcmB, were found in the noncovalently bound SLAP fractions of the S-layer-forming L. crispatus ST1, *L. amylovorus* GRL1112, and L. helveticus CNRZ32, but were not found in the non-S-layer-forming species tested (10).

There are two predicted β-*N*-acetylglucosaminidases in L. acidophilus NCFM, AcmA and AcmB. Because AcmA does not have a signal peptide sequence and has lower transcriptional expression than the other autolysins, AcmB appears to be the principal β-*N*-acetylglucosaminidase for L. acidophilus NCFM. To elucidate the role of AcmB in cell wall physiology and autolytic function, a Δ*acmB* isogenic mutant was created and complemented with an AcmB expression vector. Results indicate that the Δ*acmB* strain presents an altered cellular morphology consisting of increased chain lengths and autoaggregation due to its altered dechaining phenotype, as well as decreased stress-induced autolysis. The phenotypes of the L. acidophilus NCFM Δ*acmB* strain are consistent with the characterization of a β-*N*-acetylglucosaminidase, Acm2, in L. plantarum WCFS1 which similarly presented an altered dechaining and autolysis phenotype (29). Collectively, these data suggest that AcmB is a functioning autolysin involved in peptidoglycan turnover and daughter cell separation during cell division. Further work is necessary to characterize the specific hydrolytic activity of AcmB in L. acidophilus NCFM.

The Δ*acmB* deletion appeared to have a pleiotropic effect on the cell wall and subsequent presentation of cell surface proteins. The AcmB mutant presented a diminished capacity for binding to mucin and the extracellular matrices (ECM) collagen, fibronectin, and laminin *in vitro*. The mechanism for this adhesion phenotype remains unclear. It is possible that AcmB may directly interact with ECM in addition to its autocatalytic activity, not unlike the moonlighting proteins enolase, GAPDH, and GroEL, which have demonstrated secondary functions in adhesion to ECM in various Lactobacillus species (50–52). It is also possible that the impaired peptidoglycan hydrolysis in the Δ*acmB* mutant results in a concomitant impairment of the presentation of extracellular proteins which are covalently or noncovalently bound to the peptidoglycan. It seems most likely, however, that the reduced adhesion phenotype is due to the increased autoaggregation that resulted from the aberrant dechaining phenotype of the Δ*acmB* strain. Relevant cell surface proteins, including aggregation-promoting factors (42), fibronectin-binding proteins (21, 53), and other adhesins (54–56), may not be as exposed for contact with ECM. This interpretation of the data is consistent with previous observations of an *acmA*-deficient mutant of Lactococcus lactis MG1363, in which aberrant dechaining resulted in reduced adhesion to glass, polystyrene, and stainless steel (57).

In conclusion, we have shown that the SLAP AcmB is an autolysin involved in cell division and daughter cell separation in L. acidophilus NCFM. AcmB has an effect, directly or indirectly, on the adhesion of L. acidophilus NCFM to mucin and ECM, an important attribute for probiotic bacteria. Orthologs of AcmB are found exclusively in S-layer-forming species of the L. acidophilus homology group. Further, many of these autolysins, including AcmB, have been identified as SLAPs in the noncovalent exoproteomes of the S-layer-forming species of the said homology group. There is a dearth of information regarding the evolutionary function of S-layers in bacteria, especially those in Lactobacillus. Here, we propose that the S-layer may function as a scaffold for multiple proteins, including autolysins. Understanding the biological roles of these autolysins offers important evolutionary insights regarding the essentiality of the S-layer, as well as physiological insights regarding cell division and peptidoglycan hydrolysis in Lactobacillus species that produce S-layers.
